# The Shrinking Blind Spot: How Freeze–Thaw Obscures Microscopic Evidence of Ante-Mortem Ecchymosis

**DOI:** 10.3390/diagnostics16030419

**Published:** 2026-02-01

**Authors:** Naomi Iacoponi, Sara Giacomelli, Emanuela Turillazzi, Marco Di Paolo

**Affiliations:** Department of Surgical Pathology, Medical, Molecular, and Critical Area, Institute of Legal Medicine, University of Pisa, 56126 Pisa, Italy; n.iacoponi2@studenti.unipi.it (N.I.); s.giacomelli10@studenti.unipi.it (S.G.); emanuela.turillazzi@unipi.it (E.T.)

**Keywords:** glycophorin, immunohistochemistry, red blood cells, freeze–thaw cycles, autopsy artifacts

## Abstract

**Background/Objectives**: Histological examination constitutes a fundamental methodology for establishing the vitality of a lesion. In cases where the corpse is preserved for an extended duration of time prior to the post-mortem evaluation, particularly if the body has undergone freezing and thawing cycles, post-mortem changes may obscure or alter evidence of traumatic injuries. Consequently, the reliability of hematoxylin and eosin (H&E) staining for the reliable detection of intralesional erythrocytes in suspected traumatic fatalities is potentially severely compromised. The primary objective of this study is to rigorously underscore the detrimental influence of freeze–thaw processes on histologic examination and to advocate the indispensable incorporation of immunohistochemical analysis, specifically employing anti-human glycophorin A antibodies, to ascertain the presence of red blood cells. **Methods**: Skin samples from 10 autopsy cases were subjected to serial freeze–thaw cycles and analyzed using anti-human Glycophorin A (GPA) immunohistochemistry staining to evaluate skin lesion vitality in freeze–thawed tissues compared to fresh controls. **Results**: Results indicated that while H&E reliability was limited to fresh tissue, anti-GPA staining remained stable across all freeze–thaw cycles. **Conclusions**: Forensic pathologists must remain acutely cognizant of the potential artifacts produced by freeze–thaw cycles. In these cases, anti-GPA staining proved to be a reliable asset for evaluating the vitality of a lesion.

## 1. Introduction

Exposure to freeze–thaw cycles is known to induce significant morphological and structural modifications in human remains, impacting both gross and histological appearance. The physical process of freezing and thawing inherently disrupts tissue architecture, leading to severe structural damage at the cellular level, often manifesting as muscle fiber contraction and myelin sheath disruption in nervous tissues [1,2]. The precise biophysical mechanism underlying these alterations is complex, though common artifacts have been identified, including ice crystal formation, extracellular fluid accumulation, cellular volume changes, known as shrinkage, and tissue splitting [3]. It is hypothesized that freezing induces micromorphological changes primarily through osmotic stress. The rate of freezing and membrane permeability dictates the primary mode of damage: rapid freezing and low membrane permeability often causes the entire tissue system to freeze rapidly, whereas slow freezing and high membrane permeability typically lead to significant cellular shrinkage as water is drawn out into the extracellular space. In the latter scenario, hard ice crystals form predominantly in the extracellular matrix. Although the overall volume of tissue cells is reduced due to shrinkage, the formation of these solid ice crystals in the extracellular compartments concurrently increases the total volume of the tissue [4]. This resultant mechanical stress can critically disrupt cell-to-cell connections.

Subsequent thawing reverses some of these effects. As ice crystals melt, the extracellular solution becomes diluted and highly hypotonic. If cells had previously shrunk, the osmotic gradient drives an influx of water, causing them to swell back toward their original size. Nonetheless, the cell-to-cell connections that were permanently disrupted during the freezing phase do not redevelop. Histologically, this process of cellular shrinkage is commonly described as “karyopyknosis”, the resultant expanded extracellular areas may be evaluated as “edema” [5]. Specifically concerning human erythrocytes, their survival during freezing is critically contingent upon the unfrozen fraction and the salt concentration. Alterations in cell volume have been demonstrably shown to exert a profound influence on erythrocyte viability and structural integrity following thawing [6].

Awareness of these freeze–thaw artifacts is therefore paramount for accurate interpretation of the vitality of a lesion in post-mortem investigations [2,7]. The assessment of wound vitality is a critical task in forensic pathology, aiming to definitively establish whether an injury was inflicted ante-mortem or post-mortem. While traditional methods, such as macroscopic examination and routine histological analysis, are used to observe signs of vital reaction (e.g., inflammatory infiltrate or hemorrhage), they are often unreliable due to limitations such as operator dependency, staining artifacts, and the non-specificity of findings. In this context, although the presence of hemorrhagic infiltration does not provide conclusive evidence of vitality, it is nevertheless regarded as a key, paradigm-setting finding in the forensic evaluation of vital lesions [8]. Immunohistochemistry (IHC) has gained importance by detecting specific cellular and molecular markers released or produced by the living organism in response to trauma, such as inflammatory mediators (e.g., cytokines, selectins, chemokines) and stress proteins [9]. The behavioral standardization of these markers is essential. They can be severely compromised by external factors such as decomposition or freezing and thawing cycles, which introduce morphological and chemical artifacts that are challenging to distinguish from true vital reactions. The aim of this paper is to delineate the specific morphological alterations caused by freezing and thawing in cadaveric skin and to systematically determine how these artifacts compromise the accurate post-mortem diagnosis of lesion vitality.

## 2. Case Report

### 2.1. Case A

A 67-year-old male cyclist sustained fatal injuries following a rear-end collision with a stationary commercial truck. The entirety of the incident was documented via surveillance footage. Due to subsequent judicial delays, the post-mortem examination was performed 28 days post-mortem, during which time the remains were subjected to two distinct cycles of freezing and thawing for prolonged preservation. The external examination revealed significant traumatic findings, including superficial facial injuries (contusions and lacerations), assessed as having a vital origin, and severe facial trauma was present, characterized by multiple bilateral fractures of the maxillo-malar complex, maxilla, nasal bones, and mandible. Internal findings included fractures of the cervical vertebrae at levels C2, C6, and C7. Notably, the spinal cord appeared macroscopically intact, without evidence of apparent direct traumatic damage. Tissue samples were collected from the upper lip, lower lip, and cervical spinal cord (levels C2, C6, and C7) for histological assessment of lesion vitality.

**Figure 1:**
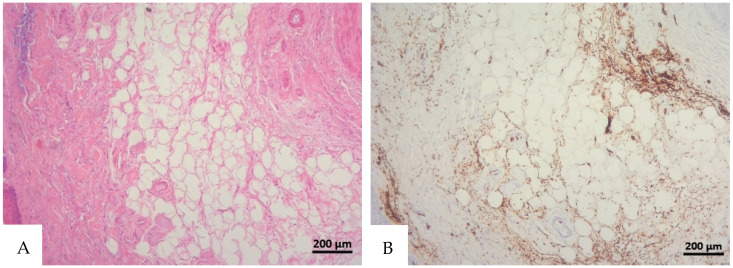
(**A**) Histological H&E staining of cutaneous tissue from a macroscopic area of discoloration in which red blood cells (RBC) cannot be identified. (**B**) Immunohistochemical staining of cutaneous tissue with anti-GPA monoclonal antibody, positive for RBC extravasation. The sample shows the presence of RBCs in some of the areas that were marked as dubious in the H&E staining (original magnification 10×).

**Figure 2:**
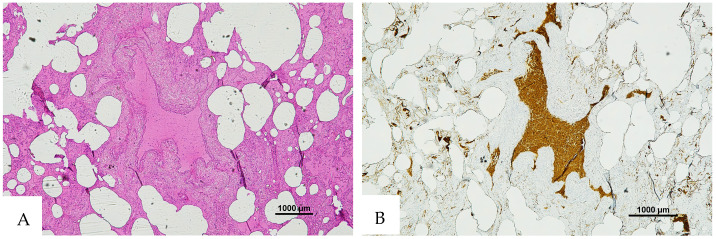
(**A**) Histological H&E staining of pulmonary tissue, in which the presence of a vessel with RBCs and eosinophilic areas is suspicious of hemorrhagic edema. (**B**) Immunohistochemical staining of cutaneous tissue with an anti-GPA monoclonal antibody. The sample is negative for RBC extravasation, showing RBCs exclusively inside vessels (original magnification 10×).

The initial histological investigation using H&E staining failed to reliably demonstrate the presence of extravasated red blood cells at the injury sites, a finding potentially interpreted as a lack of vital reaction (Figure 1A and Figure 2A). This absence was critically attributed to the severe cellular degradation and osmotic damage induced by the two freeze–thaw cycles. To definitively confirm the vital nature of the observed lesions—a distinction essential for forensic reconstruction—immunohistochemical staining with an anti-human Glycophorin A antibody was employed. The strong positivity observed across all sampled tissues (lips, cervical spinal cord and lung) unequivocally confirmed the presence of ante-mortem hemorrhage (Figure 1B and Figure 2B).

### 2.2. Case B

An elderly female was found dead inside her residence. Initially, the circumstantial information and evidence gathered framed the case as among deaths due to natural causes. During a preliminary inspection of the body, however, we noticed multiple suspicious ecchymoses on the woman’s upper limbs and face, along with a mark on the right upper limb compatible with acupuncture. Samples of these lesions were collected for histological examination. The autopsy was then suspended due to criminal procedural issues, and the corpse was stored in a refrigerated environment at −20 °C. The post-mortem examination was then completed after more than a month and revealed the presence of further dubious areas of reddish coloration. We proceeded to collect multiple tissue samples for histological investigation.

The suspected ecchymotic areas collected during the preliminary assessment were subjected to routine H&E staining, which unequivocally confirmed the presence of extravasated red blood cells.

**Figure 3:**
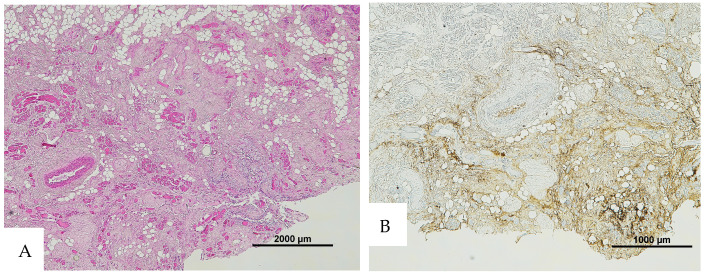
(**A**) Histological H&E staining of cutaneous tissue from a macroscopic area of discoloration sampled during the second phase of the autopsy, suggestive of RBC extravasation. (**B**) Immunohistochemical staining of cutaneous tissue with anti-GPA monoclonal antibody, positive for RBC extravasation. The sample shows the presence of RBCs in some of the areas that were marked as dubious in the H&E staining (original magnification 4×).

Conversely, samples collected after the freeze–thaw cycle were negative for red blood cells with H&E staining. The immunohistochemical staining with anti-GPA antibody was then employed, with positivity in most of the samples (Figure 3).

## 3. Materials and Methods

Accurately distinguishing between vital (ante-mortem) and post-mortem lesions is fundamental and critical in forensic autopsies and criminal investigations. The initial step in this assessment is to determine the presence of erythrocyte extravasation in the surrounding tissues. This is conventionally performed using hematoxylin and eosin (H&E) staining. However, in cadavers subjected to freeze–thaw cycles, this standard technique may fail to reliably detect such extravasation.

To establish a standardized approach for evaluating lesions in such modified tissue, we identified 10 autopsy cases involving traumatic deaths, and from each case, skin samples displaying macroscopic ecchymosis were harvested. We employed both histochemical and immunohistochemical staining techniques to assess lesion vitality. An anti-GPA monoclonal antibody was used as an indirect marker for red blood cells. This specificity is crucial, as it avoids the diffusion artifacts common with markers such as hemoglobin, which can passively diffuse from blood vessels into surrounding tissues, creating false positives.

Each specimen was divided into four portions, representing distinct processing groups. Portion 1 was fixed in 10% formalin, sliced, and stained with hematoxylin and eosin, constituting the baseline Group 1, time 0) of fresh, unfrozen tissue. Portion 2 was sampled after the tissue was frozen at −20 °C for 15 days and then thawed. This tissue ribbon was processed identically to Group 1 and stained with an anti-GPA monoclonal antibody, defining Group 2 (time T1, after one freeze–thaw cycle). After an additional 15-day interval, the same tissue underwent a second freeze–thaw cycle; a third ribbon was then processed and stained with both H&E and anti-GPA monoclonal antibody, forming Group 3 (time T2, after two freeze–thaw cycles). To validate the diagnostic specificity of the anti-GPA antibody and confirm the absence of non-specific binding, an additional sample of adjacent, macroscopically undamaged skin tissue was processed identically to the experimental groups and utilized as a negative tissue control (Group 4).

All sections were examined macroscopically, and the diagnostic reliability of H&E versus GPA staining was systematically compared across the three temporal points (T0, T1, and T2).

Due to the pilot nature of this investigation, the experimental design prioritized high intra-sample reproducibility, utilizing multiple standardized sections from each specimen to ensure the consistent performance of the GPA marker across repeated freeze–thaw cycles.

## 4. Results

Histological examinations using H&E staining were initially performed to microscopically assess the presence and extent of red blood cell extravasation, a classic indicator of lesion vitality.

**Figure 4:**
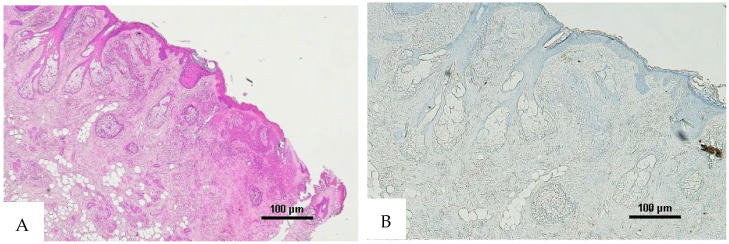
(**A**) Histological H&E staining of cutaneous tissue of Group 4, showing the presence of a vessel with RBCs and areas suspicious for RBC extravasation. (**B**) Immunohistochemical staining of cutaneous tissue with an anti-GPA monoclonal antibody. The sample is negative for RBC extravasation, showing RBCs exclusively inside vessels (image A original magnification 4×, image B original magnification 10×).

**Figure 5:**
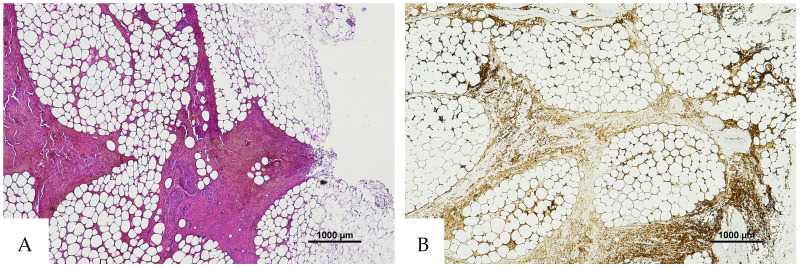
(**A**) Histological H&E staining of the cutaneous tissue of Group 0, positive for RBC extravasation. (**B**) Immunohistochemical staining of cutaneous tissue with anti-GPA monoclonal antibody, positive for RBC extravasation (original magnification 10×).

**Figure 6:**
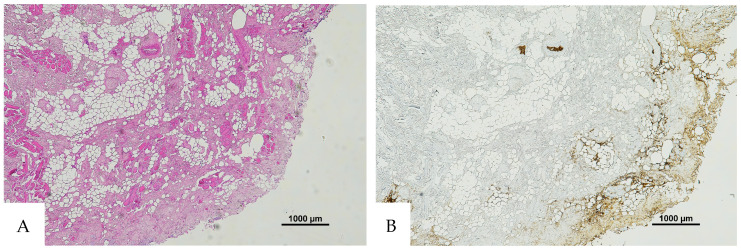
(**A**) Histological H&E staining of the cutaneous tissue of Group 1, dubious for RBC extravasation. Some areas show the presence of eosinophilic pigment. (**B**) Immunohistochemical staining of cutaneous tissue with anti-GPA monoclonal antibody, positive for RBC extravasation. The sample shows the presence of RBCs in some of the areas that were marked as dubious in the H&E staining (original magnification 4×).

**Figure 7:**
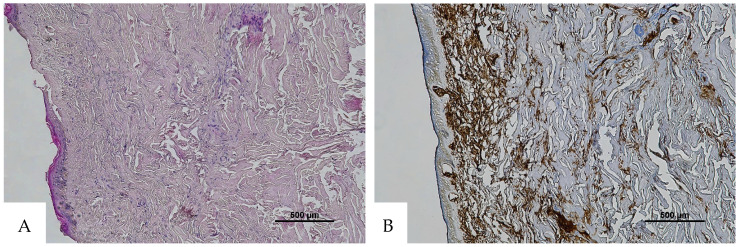
(**A**) Histological H&E staining of the cutaneous tissue of Group 2, in which RBCs cannot be identified. (**B**) Immunohistochemical staining of cutaneous tissue with an anti-GPA monoclonal antibody. The sample shows extensive presence of RBC extravasation (original magnification 20×).

**Figure 8:**
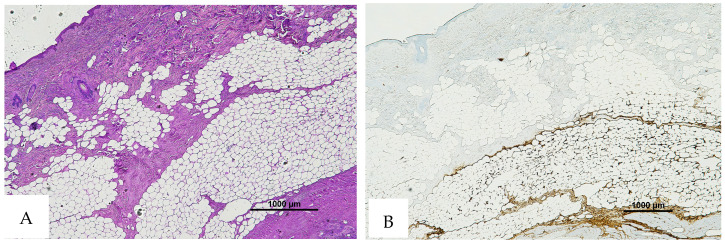
(**A**) Histological H&E staining of cutaneous tissue of Group 2, in which RBCs cannot be identified. (**B**) Immunohistochemical staining of cutaneous tissue with an anti-GPA monoclonal antibody. The sample shows extensive presence of RBCs extravasation (original magnification 4×).

In the control group (T0), which comprised fresh, unfrozen tissue samples, H&E staining unambiguously demonstrated diffuse hemorrhagic infiltration within the dermal layer across all investigated specimens. This confirmed the baseline presence of ante-mortem trauma. The analysis of samples subjected to a single freeze–thaw cycle (T1) revealed a notable degradation in the diagnostic reliability of H&E staining, evidenced by diminished or equivocal visualization of extravasated erythrocytes compared to the control. Crucially, however, the immunohistochemical analysis using the anti-human GPA monoclonal antibody yielded unequivocally positive results, albeit indirectly, for RBCs in all Group 2 samples. The impact of the artifacts became pronounced in tissues exposed to two freeze–thaw cycles (T2). In this cohort, H&E staining was rendered unreliable, often failing to detect or unambiguously identify the presence of red blood cells due to severe cellular degradation and morphological alterations. Despite this significant post-mortem artifact, the specificity and resilience of the GPA staining technique persisted, as it yielded positive detection of RBC membrane material in every sample tested. The comparative analysis of H&E and GPA staining is illustrated in Figure 4, Figure 5, Figure 6, Figure 7, Figure 8, while Table 1 provides a comprehensive summary of the results. 

## 5. Discussion

The accurate distinction between vital and post-mortem lesions remains a cornerstone of forensic pathology and a determinant in criminal investigations. The accuracy of this determination not only directs the reconstruction of events but also profoundly influences the final legal judgement. In routine practice, the approach still relies on macroscopic examination and routine histological analysis, which search for classic signs of vital reaction (e.g., inflammatory infiltrate, fibrin deposition, or collagen retraction). However, as widely acknowledged in the literature, these methods exhibit significant limitations: standard histology is prone to operator dependency, potential staining artifacts, and critically, the non-specificity of findings, as similar alterations can sometimes manifest in non-vitally injured tissues. To overcome these deficiencies, modern forensic pathology has integrated more sensitive and specific techniques. Immunohistochemistry, based on antigen-antibody reactions, has taken a prominent role by enabling the localization and quantification of molecular markers released in response to trauma, such as inflammatory mediators (cytokines, chemokines) and cellular stress proteins [9,10].

A significant and often underappreciated complication arises when cadavers are subjected to freeze–thaw cycles prior to autopsy. This process has been demonstrated to induce profound histological artifacts and, therefore, false negatives that can effectively obscure or eliminate critical investigative evidence. In cases of violent death, the capacity to confirm a hemorrhagic lesion’s vital origin is pivotal to directing the course of the forensic investigation. The unreliability of the H&E staining in these compromised corpses is hypothesized to result from severe shrinkage and structural disruption of erythrocyte microstructures caused by osmotic stress during the freeze–thaw process.

While the literature addressing the histological artifacts of freeze–thaw cycles is not as extensive as desirable, significant alterations have been consistently reported. Freezing induces major changes, including the loss of cellular affinity for routine stains, extracellular fluid accumulation, cellular shrinkage, tissue clefts/fractures, hemolysis, and hematin formation. More subtle alterations include the loss of bronchial cilia and the increased prominence of collagen in alveolar septa and meninges [3,11]. The most consistently observed artifacts are the expansion of extracellular spaces and pronounced cell shrinkage. Organs with high fluid content and cellular density, such as the heart and liver, are often the most significantly impacted. Generally, strong basophilic nuclear staining and hemolysis are widespread across most tissues [12,13].

We investigated two cases in which macroscopic examination showed the presence of ecchymosis and excoriations. However, routine histological investigation of the skin lesions failed to identify the presence of red blood cells in the subcutaneous tissue. Microscopic examination of these samples revealed characteristic freeze–thaw artifacts, including extracellular space expansion, tissue clefts, and cellular shrinkage. Furthermore, karyopyknosis and vacuolization of the epidermis, consistent with observations by Tabata et al., were evident in the H&E-stained sections [14]. The unequivocal demonstration of the diagnostic limitations of routine hematoxylin and eosin staining for evaluating wound vitality due to cryopreservation artifacts in this case provided the rationale for an experimental study. This subsequent research aimed to define and standardize the application of the Glycophorin A immunomarker as a robust and specific alternative for identifying erythrocytes in samples compromised by repeated freeze–thaw cycles.

To overcome the diagnostic limitations-imposed freeze–thaw artifacts, immunohistochemical staining with an anti-human GPA monoclonal antibody should be used. Glycophorin (GPA, GPB, GPC, and GPD) is a family of transmembrane proteins that constitute approximately 2% of the total RBC membrane protein mass, with GPA being the predominant species. GPA is an ideal marker because its localization to the cell membrane provides high specificity for erythrocyte remnants, unlike cytoplasmic markers (e.g., hemoglobin), which can diffuse into surrounding tissue following cell rupture. Clinical medicine uses GPA antibodies extensively to identify erythroid precursors, and this application is highly transferable to forensic pathology for the reliable identification of RBCs in compromised tissues [15].

Previous forensic studies have validated the utility of immunohistochemistry in bodies subjected to tissue-altering conditions. Research on corpses dying from hypothermia demonstrated that certain antigen identification remains possible despite tissue alteration [3,11,12]. Specifically, Baldari et al. confirmed that anti-human GPA antibodies effectively detect RBCs across different post-mortem intervals, indicating robustness against general degradation [16]. Furthermore, Cattaneo et al. successfully employed GPA antibodies to identify clots and RBC residues on bone fracture margins, establishing a positive GPA reaction as strongly indicative of a vital reaction [17]. Consequently, anti-human GPA antibodies are increasingly recognized as a reliable marker for investigating erythrocytic residues in poorly preserved or compromised tissues.

In alignment with the current literature, our study provides concrete evidence that immunohistochemical analysis using anti-human Glycophorin A antibodies successfully identified hemorrhage in skin samples after both one and two freeze–thaw cycles. This finding is significant, given the limited research specifically detailing the effect of freeze–thaw cycles on erythrocyte integrity in situ. Ishiguro et al.’s work on RBCs in saline demonstrated rupture due to freezing damage, supporting the physical mechanism that leads to the disappearance of visible red blood cells in H&E sections [18].

The practical necessity for this research is highlighted by the procedural technicalities within judicial systems, such as the Italian system, which may mandate repeated cycles of freezing and thawing—particularly in medical malpractice judicial autopsies—to ensure meticulous evidence preservation prior to expert examination. Forensic experts must therefore be acutely aware of the severe freeze–thaw phenomenon and the pivotal diagnostic superiority of IHC with GPA in these challenging cases.

### Limitations of the Study

Despite the promising results observed in cutaneous samples, this study is primarily limited by its exclusive focus on skin tissue. While the efficacy of GPA immunohistochemical staining has been demonstrated on our sample groups, the findings cannot yet be generalized to the entire post-mortem examination. Forensic practice is based on the evaluation of multiple organ tissues; however, the diagnostic performance of GPA in other internal structures remains unexamined in this context. Specifically, organs with high metabolic activity or specific histological architectures, such as the heart and liver, are known to be significantly more susceptible to freeze–thaw artifacts and rapid autolysis. The applicability of this GPA protocol to those tissues is still purely theoretical. Moreover, the study examined only ten cases, which limits statistical power and the ability to capture the full spectrum of post-mortem variability.

Further empirical research is therefore essential to validate this marker across different tissues, a necessary step toward establishing a comprehensive and standardized diagnostic protocol for severely compromised forensic specimens.

## 6. Conclusions

The results of the present study have the potential to make significant contributions to the field of forensic pathology. Procedural technicalities may necessitate that a corpse undergo repeated cycles of freezing and thawing prior to an autopsy. In the event of prolonged preservation, post-mortem changes have the potential to obscure evidence of traumatic injuries. The use of immunohistochemical staining using an anti-human Glycophorin A antibody to detect the presence of red blood cells is therefore critical in such investigations.

## Figures and Tables

**Table 1:** This table summarizes the results of the experimental study. The 10 rows of subjects represent the 10 individual cases used in this study.

Subject	T0 (H&E)	T1 (H&E)	T1 (GPA)	T2 (H&E)	T2 (GPA)
1	Positive	Negative	Positive	Negative	Positive
2	Positive	Inconclusive	Positive	Negative	Positive
3	Positive	Negative	Positive	Negative	Positive
4	Positive	Negative	Positive	Negative	Positive
5	Positive	Inconclusive	Positive	Negative	Positive
6	Positive	Negative	Positive	Negative	Positive
7	Positive	Inconclusive	Positive	Negative	Positive
8	Positive	Negative	Positive	Negative	Positive
9	Positive	Negative	Positive	Negative	Positive
10	Positive	Inconclusive	Positive	Negative	Positive

## Data Availability

The original contributions presented in this study are included in the article. Further inquiries can be directed to the corresponding author.

## Abbreviations

The following abbreviations are used in this manuscript:

GP	Glycophorin
H&E	Hematoxylin and Eosin
IHC	Immunohistochemistry
RBC	Red Blood Cell
T	time
